# TRPM8 and Na_v_1.8 sodium channels are required for transthyretin-induced calcium influx in growth cones of small-diameter TrkA-positive sensory neurons

**DOI:** 10.1186/1750-1326-6-19

**Published:** 2011-03-04

**Authors:** Robert J Gasperini, Xu Hou, Helena Parkington, Harry Coleman, David W Klaver, Adele J Vincent, Lisa C Foa, David H Small

**Affiliations:** 1 Menzies Research Institute, University of Tasmania, Tasmania, 7001, Australia; 2 School of Medicine, University of Tasmania, Tasmania, 7001, Australia; 3 Howard Florey Institute, University of Melbourne, Victoria, 3010, Australia; 4 Department of Physiology, Monash University, Victoria, 3800, Australia

## Abstract

**Background:**

Familial amyloidotic polyneuropathy (FAP) is a peripheral neuropathy caused by the extracellular accumulation and deposition of insoluble transthyretin (TTR) aggregates. However the molecular mechanism that underlies TTR toxicity in peripheral nerves is unclear. Previous studies have suggested that amyloidogenic proteins can aggregate into oligomers which disrupt intracellular calcium homeostasis by increasing the permeability of the plasma membrane to extracellular calcium. The aim of the present study was to examine the effect of TTR on calcium influx in dorsal root ganglion neurons.

**Results:**

Levels of intracellular cytosolic calcium were monitored in dorsal root ganglion (DRG) neurons isolated from embryonic rats using the calcium-sensitive fluorescent indicator Fluo4. An amyloidogenic mutant form of TTR, L55P, induced calcium influx into the growth cones of DRG neurons, whereas wild-type TTR had no significant effect. Atomic force microscopy and dynamic light scattering studies confirmed that the L55P TTR contained oligomeric species of TTR. The effect of L55P TTR was decreased by blockers of voltage-gated calcium channels (VGCC), as well as by blockers of Na_v_1.8 voltage-gated sodium channels and transient receptor potential M8 (TRPM8) channels. siRNA knockdown of TRPM8 channels using three different TRPM8 siRNAs strongly inhibited calcium influx in DRG growth cones.

**Conclusions:**

These data suggest that activation of TRPM8 channels triggers the activation of Na_v_1.8 channels which leads to calcium influx through VGCC. We suggest that TTR-induced calcium influx into DRG neurons may contribute to the pathophysiology of FAP. Furthermore, we speculate that similar mechanisms may mediate the toxic effects of other amyloidogenic proteins such as the β-amyloid protein of Alzheimer's disease.

## Background

Protein misfolding is a common feature of many neurodegenerative diseases. In some of these diseases, such as the synucleinopathies and the tauopathies, cytoplasmic proteins aggregate to form intracellular deposits. However, in the amyloidoses, which include Alzheimer's disease (AD), prion diseases and the British and Danish familial dementias, proteinaceous aggregates are observed extracellularly [1-4]. There is increasing evidence that the mechanism of neurotoxicity in these amyloidoses is similar and that it is the conformation of the aggregated protein, rather than its specific amino acid sequence which results in altered membrane permeability to calcium [5]. Therefore, studies on the mechanism of neurotoxicity in one disease may provide insights into the mechanisms involved in other diseases.

Familial amyloidotic polyneuropathy (FAP) is a rare autosomal dominant disease characterised by the deposition of transthyretin (TTR) protein in peripheral nerves. The early clinical manifestations of FAP include progressively aberrant thermosensation and nociception in the lower extremities followed by profound autonomic dysfunction [6-9]. TTR is a 55 kD homotetrameric protein that has been well characterised for its role in the transport of thyroxine and retinol [8]. More than 100 TTR mutations are known, and most have been shown to be amyloidogenic [10]. Many studies have shown that mutant TTR aggregates to form oligomers more readily than wild-type TTR, and that further aggregation leads to the formation of amyloid fibrils [11]. There is a correlation between the rate of aggregation of TTR in vitro and the extent or severity of the disease phenotype. For example, the rare L55P mutation produces a more aggressive amyloidosis than the more common V30M mutation, and *in-vitro *studies show that L55P TTR aggregates more much readily than V30M TTR [12-16].

The mechanism by which TTR forms fibrils is not entirely understood. Some studies suggest that amyloid deposition involves the formation of low molecular weight "nuclei" that must reach a critical concentration before fibril elongation [17]. However, other studies suggest that amyloid aggregation may be a nucleation-independent process [18,19]. More specifically, and consistent with this latter view, Hammarström *et al *[20] and Hurshman Babbes *et al *[14] have shown that TTR aggregation may be a nucleation-independent process. Mutant TTR has been shown to be toxic to cells in culture [12,21]. It has been reported that TTR-induced toxicity is mediated by the receptor for advanced glycation end-products (RAGE) and that activation of RAGE leads to endoplasmic reticulum stress, activation of ERK1/2 and caspase-dependent apoptosis [22]. There is also evidence to suggest that misfolded proteins like TTR mediate their toxic effects by binding directly to lipid-rich areas of the plasma membrane [13,23]. Also, the toxicity of TTR aggregates is correlated with membrane binding affinity, destabilisation of cell membrane fluidity and subsequent decrease in cell viability [13].

There is ample evidence suggesting that some of the toxic effects of amyloid proteins are mediated via an increase in calcium permeability. For example, the β-amyloid protein (Aβ) of AD is known to induce calcium influx into cells [24,25]. This disruption of calcium homeostasis is likely to cause abnormal neuronal function since calcium is an important mediator of synaptic plasticity and excitotoxicity. However, the mechanism by which amyloid proteins induce calcium entry in cells is poorly understood.

Previously, we have shown that in SH-SY5Y neuroblastoma cells, TTR induces an influx of extracellular calcium across the plasma membrane. This TTR-induced increase in calcium permeability is primarily mediated by voltage-gated calcium channels (VGCC), with a small proportion (~20%) of the calcium influx through voltage-independent channels [12]. However, neuroblastoma cells are not a physiologically relevant cell type for studying FAP. FAP is a peripheral polyneuropathy involving amyloid deposition affecting peripheral neurons including sensory neurons of dorsal root ganglia (DRG).

In the present study, we examined the effect of amyloidogenic forms of TTR on calcium levels in cultured DRG neurons. We demonstrate that TTR induces calcium influx into DRG neurons, similar to that observed using SH-SY5Y cells. Importantly, we demonstrate that calcium influx into the growth cones of small-diameter TrkA-positive DRG neurons requires the presence of Na_v_1.8 voltage-gated sodium channels and transient receptor potential (TRP) M8 channels. The results suggest that activation of TRPM8 channels by L55P TTR results in the subsequent opening of voltage-sensitive sodium and calcium channels. Our study suggests that TRP channels may be an important therapeutic target, not only for the development of drugs for the treatment of FAP, but also for many important neurodegenerative diseases.

## Results

### Effect of TTR on calcium

Previously, we showed that the amyloidogenic TTR protein variant L55P caused a significant calcium influx into SH-SY5Y neuroblastoma cells [12]. These findings led us to examine whether calcium dysregulation was a feature of L55P-induced toxicity in sensory neurons, which are a pathophysiologically relevant cell system. Initially, we examined whether L55P could elicit a calcium response in DRG neurons. DRG growth cones are highly accessible structures and easily imaged using routine techniques. They express a variety of receptors and ion channels, crucial for a variety of autonomous signalling mechanisms involved in motility [26], cytoskeletal rearrangements [27] and transduction of guidance molecules [28]. Significantly, calcium is a key second messenger molecule involved in the transduction all of these processes. To examine the effect of TTR on calcium in DRG, we used single-wavelength calcium imaging with the cell-permeable calcium indicator Fluo-4 AM and monitored fluorescence responses after the application of TTR (0.5 mg/ml) to the incubation medium.

**Figure 1:**
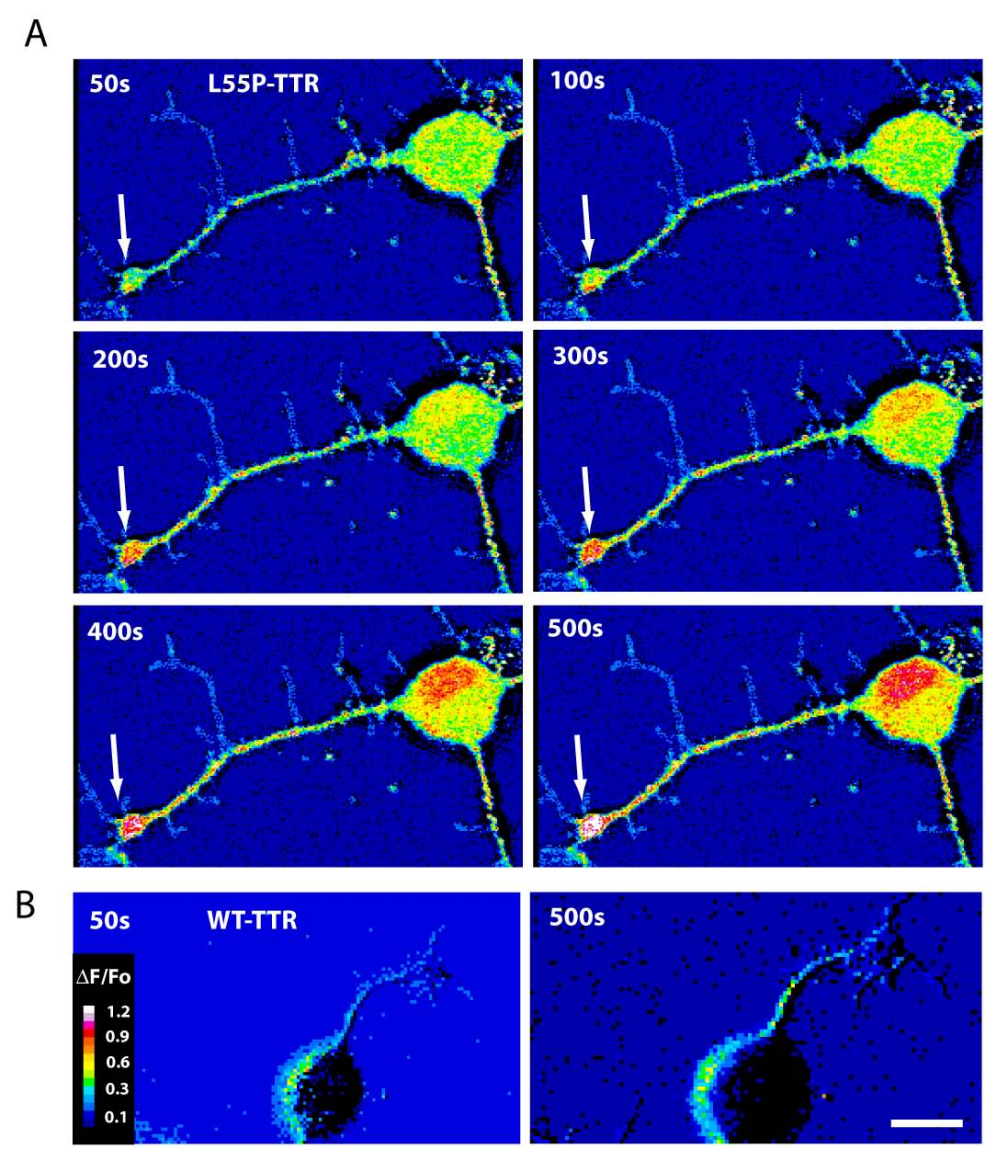
**Effect of TTR on calcium**. Cultures of DRG neurons were treated with L55P (A) or wild-type (B) TTR and intracellular calcium flux (ΔF/F_0_) was measured over a 500 sec time course. Addition of L55P elicited greater calcium fluxes than WT. Significantly, calcium entry at growth cones (arrows) occurred earlier than at DRG cell soma. Scale bar is 5 μm.

**Figure 2:**
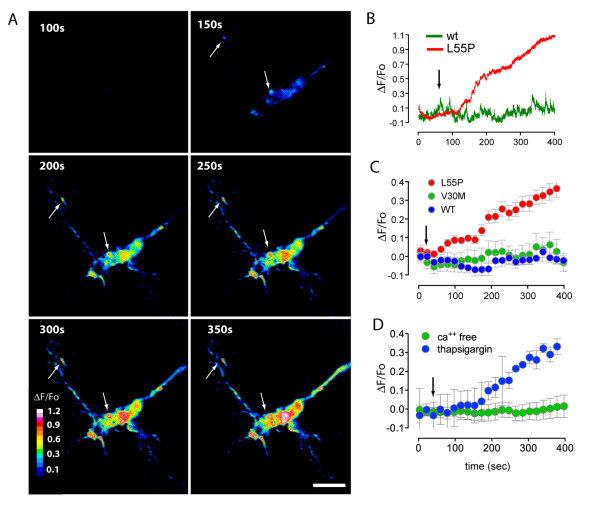
**Dynamics of extracellular calcium influx**. (A) Single wavelength calcium imaging of isolated DRG growth cones revealed distinct spatio-temporal kinetics of calcium flux (ΔF/F_0_), in growth cones. Calcium signals were not homogeneous in the central area and extended out to filipodial tips and lamelipodial leading edges (arrows). (B) Example of growth cone calcium responses to L55P treatment compared to WT protein added to imaging buffer (arrow) (C-D) Pooled results of calcium fluorescence after TTR addition (arrow). L55P (0.5 mg/ml, n = 18) elicited a sustained increase in fluorescence when compared with WT (0.5 mg/ml, n = 15) (B), fresh and V30M (0.5 mg/ml, n = 16). (D) L55P calcium influx in DRG growth cones was derived from extracellular sources. L55P addition did not elicit a significant calcium increase when cells were imaged in calcium-free buffer (n = 8). Significant L55P-induced calcium influx was seen in growth cones following treatment with thapsigargin (150 nM) to deplete calcium stores (n = 12). Scale bar is 5 μm. Error bars indicate mean ± SEM.

Between 12-24 hr after plating, in the presence of NGF, DRG cultures contained predominantly small-diameter (<15 μm) neurons with extensive axons and large, effusive growth cones. Larger diameter neurons which are typically observed *in vivo *were less numerous in culture and typically were devoid of growth cones and were not imaged. After addition of freshly prepared L55P to the culture medium, a sustained increase in the cytosolic calcium concentration ([Ca^2+^]_i_) was observed in many neurons. [Ca^2+^]_i _was observed to increase initially in distal growth cones approximately 100 sec after addition of L55P to the culture medium (Figure 1A, arrows), and then this increase in [Ca^2+^]_i _in growth cones was followed by a rise in [Ca^2+^]_i _in the cell soma, approximately 100-200 sec after addition of L55P. No increase in [Ca^2+^]_i _was observed when WT protein was added to cultures (Figure 1B and 2B).

As the rise in [Ca^2+^]_i _was observed initially in the growth cones, we focussed our attention on the effect of TTR on growth cone calcium dynamics. A closer examination of growth cones revealed a distinct spatio-temporal pattern of calcium fluorescence. Initially, discrete areas of increased calcium were detected at filipodial tips and lamellipodial leading edges (Figure 2A, arrows). Within 300 sec of L55P addition, regions of increased calcium flux were evident over the entire growth cone. Addition of freshly prepared V30M protein had little effect on [Ca^2+^]_i_, (Figure 2C). However, when V30M was aged for 36 hr, a small calcium influx was observed (see Additional file 1). These results are consistent with our previous calcium imaging experiments using SH-SY5Y cells [12] which showed that aged V30M induced calcium influx and was toxic to the cells in vitro.

To determine whether the rise in cytoplasmic calcium originated from extracellular or intracellular sources, growth cones were exposed to L55P and imaged in calcium and magnesium-free imaging medium containing 300 μM EGTA. This treatment completely abolished the sustained rise in [Ca^2+^]_i _seen in complete media (Figure 2D). We also examined the effect of depleting endoplasmic reticulum (ER) calcium stores on the L55P-induced increase in [Ca^2+^]_i _by treating cultures with thapsigargin (150 nM for 30 min) prior to imaging. Thapsigargin did not significantly reduce the calcium influx (Figure 2D). Taken together, these data suggest that the increase in [Ca^2+^]_i _by L55P in DRG originates from extracellular sources.

### Analysis of aggregation state

**Figure 3:**
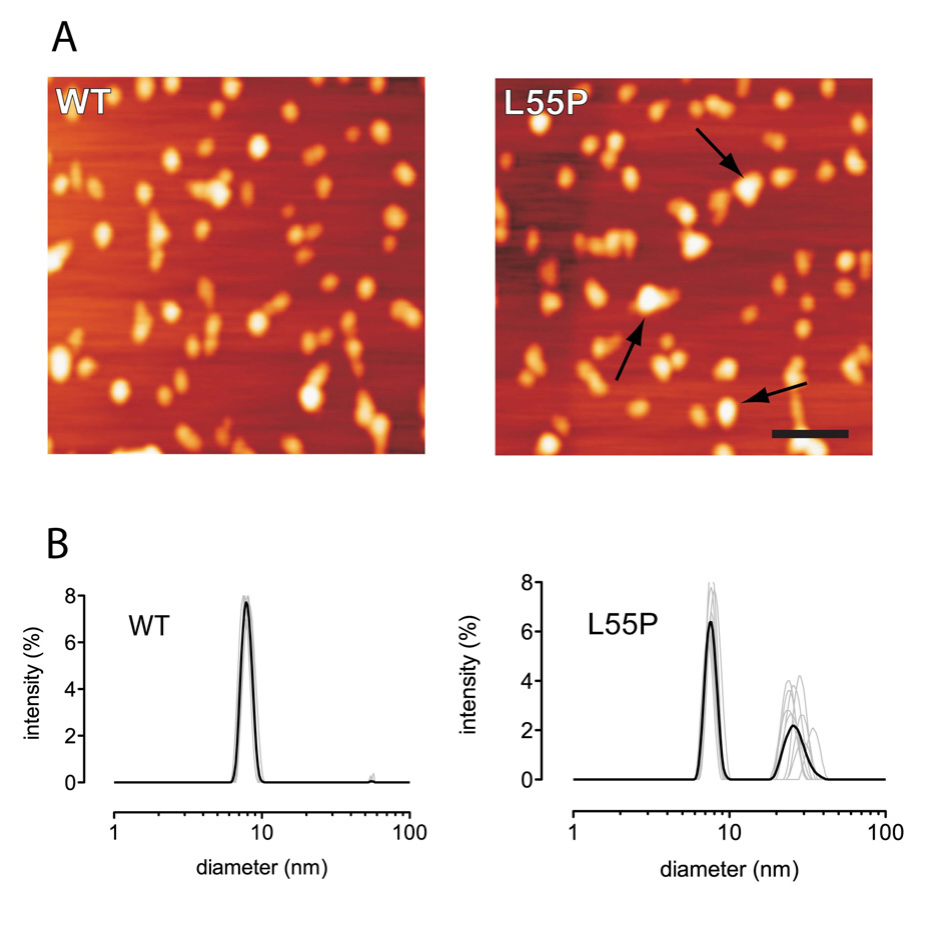
**Analysis of TTR aggregation by AFM and dynamic light scattering**. (A) Freshly prepared WT and L55P were examined using AFM. Representative AFM images show a relatively homogeneous dispersion of non-fibrillar particles in both WT and L55P preparations. Image analysis of particle cross-sectional area revealed a distribution of significantly larger L55P particle sizes (arrows) compared with those of freshly prepared WT protein (WT TTR 162 ± 63 nm^2 ^compared with L55P TTR 263 ± 96 nm^2^, n = 86). Freshly prepared TTR protein was also examined using dynamic light scattering. (B) Protein samples were measured repetitively (replicates analyses in grey, mean of replicates in heavy black line) in a DLS every 30 min over a 4-6 hr time period by DLS. In addition to the native tetrameric molecule (7.66 ± 0.56 nm) corresponding to an estimated MW of 78 ± 5.7 kDa, a significant oligomeric species (29.38 ± 2.59 nm) corresponding to an estimated MW of 1810 ± 159.8 kDa, was detected in L55P samples compared to WT samples. Large molecular mass aggregates (>1000 nm in diameter) were not detected in protein preparations during the 4-6 hr analysis period. Scale bar is 100 nm.

As L55P induced calcium influx and WT did not, we examined the aggregation state of the two preparations used for the calcium imaging studies (Figure 3). Freshly prepared WT and L55P were first analysed by atomic force microscopy (AFM) (Figure 3A). Using this method, both forms of TTR contained predominantly globular or amorphous particles ranging in apparent size from approximately 10-50 nm in diameter. No fibrillar structures were observed. The calculated diameter of a native TTR tetramer is approximately 7 nm. Therefore, the smallest particles (10 nm) were probably tetramers, as tip convolution effects lead to an overestimation of the size of objects in the XY plane. As a single AFM field did not reveal dramatic differences in particle size distribution between the WT and L55P forms of TTR, quantitative image analysis of AFM particle cross-sectional area was also performed. This revealed that average apparent particle sizes of L55P (263 ± 96 nm^2^, n = 86) were greater than those of the WT preparation (162 ± 63 nm^2^, n = 52).

To examine the state of aggregation of TTR preparations further and to obtain a more accurate measure of particle size distribution, both preparations were also analysed by dynamic light scattering (DLS) (Figure 3B). WT showed a discrete peak intensity corresponding to a diameter of 7.7 nm ± 0.6 nm, whereas L55P showed an additional peak intensity corresponding to a particle size of 29.4 nm ± 2.6 nm. Assuming a globular structure and a specific density of 1.39 g/cm^3^, which would be typical for many proteins, this size would correspond to a molecular mass of 1810 ± 160 kDa. This molecular species would therefore be predicted to contain approximately 130 TTR monomers.

### Effect of ion channel blockers

As voltage-gated calcium channels (VGCC) were previously found to mediate calcium entry in SH-SY5Y cells [12], and as DRG sensory neurons express a variety of VGCCs [29], we examined whether L55P could influence calcium permeability through a similar mechanism at DRG growth cones.

**Figure 4:**
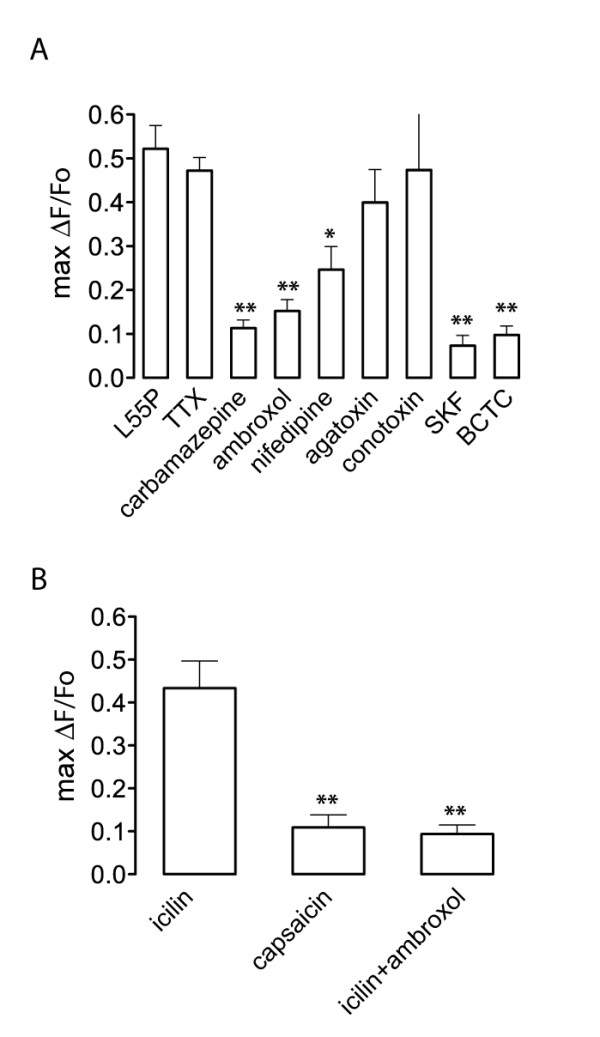
**Effect of ion channel blockers on L55P TTR-induced calcium influx and analysis of sensitivity to icilin and capsaicin**, (A) L55P was applied to DRG growth cones in the presence of VGCC inhibitors (nifedipine, ω-agatoxin IVA, ω-conotoxin GIVA), Na_V _inhibitors (tetrodotoxin, ambroxol and carbamazepine) and TRP inhibitors (SKF-96365, BCTC). The resulting maximal calcium influx (max ΔF/F_0_) calculated over the imaging period (7 min) was calculated. (B) Effect of capsaicin (1 μM) and icilin (100 μM) on cytosolic calcium in DRG growth cones in culture. When DRG cultures were pre-treated with ambroxol (5 μM), icilin-induced calcium fluorescence was significantly decreased. All graphs show maximal ΔF/F_0 _± SEM for n = 12-24 growth cones. Significant differences from control values are depicted as: * p < 0.05; **p < 0.005; Mann-Whitney U-test. Error bars indicate mean ± SEM.

First, we examined the involvement of VGCC in TTR-induced calcium influx (Figure 4A). The L-type VGCC blocker nifedipine (5 μM) blocked L55P-induced calcium influx approximately 50%. Although mean levels of calcium influx were lower in the presence of the P-type VGCC inhibitor, ω-agatoxin IVA (1 μM) or the N-type VGCC inhibitor ω-conotoxin GVIA (1 μM), these differences were not statistically significant. To examine the possibility that the activation of L-type VGCC by L55P might be due to a change in membrane potential, we examined the effect of voltage-gated sodium channel (Na_V_) blockers. Tetrodotoxin (TTX) (2 μM) did not significantly reduce the magnitude of L55P-induced calcium influx. However, the lack of effect of TTX was not unexpected since TTX-resistant (TTX_R_) Na_V _channels (Na_V_1.8) predominate in DRG neurons [30,31]. Therefore, we also used two relatively specific inhibitors of TTX_R _channels, ambroxol (5 μM) and carbamazepine (5 μM). Blockade of Na_V_1.8 channels significantly reduced TTR-induced calcium influx by 67% and 74%, respectively, indicating that activation of TTX_R _sodium channels was necessary for L55P-induced calcium influx.

Since blockade of voltage-gated channels failed to completely block calcium entry, we explored the possibility that the remaining calcium influx was mediated by voltage-insensitive channels. The transient receptor potential (TRP) cation channels are important mediators of sensory stimuli in the peripheral nervous system [32]. To determine the involvement of TRP channels in growth cone calcium entry following treatment with TTR, we used a range of TRP channel blockers with various specificities for inhibiting specific classes of TRP channels. In support of the view that TRP channels mediate TTR-induced calcium entry, we found that SKF96365 (5 μM), an inhibitor of TRPC [33] and TRPM8 channels [34], strongly blocked L55P-induced calcium entry in DRG growth cones. Interestingly, 5 μM BCTC, an antagonist of TRPV1 and TRPM8 channels [35] also decreased the L55P-induced calcium influx in growth cones from small-diameter DRG neurons.

**Figure 5:**
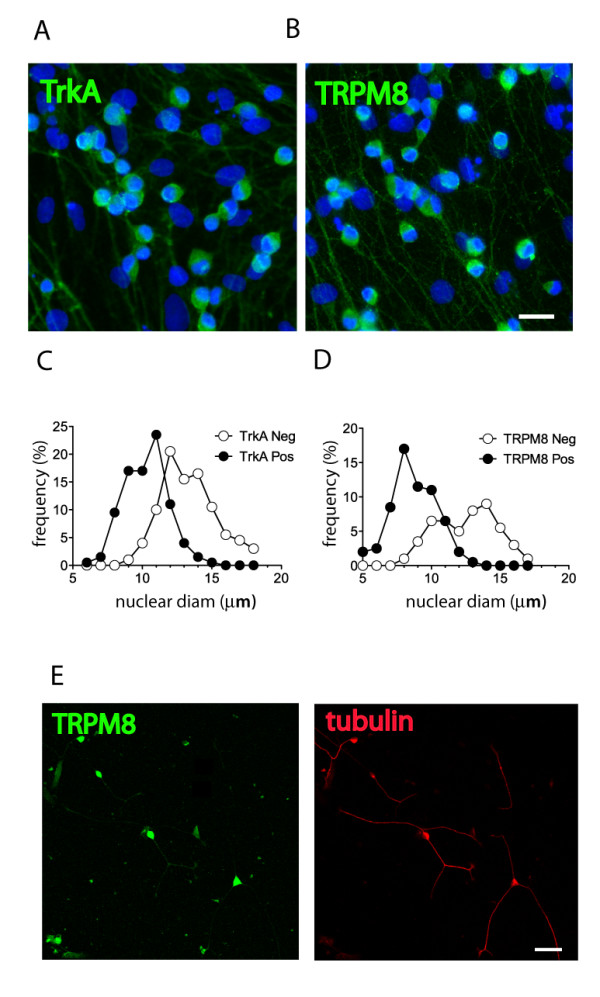
**TrkA- and TRPM8 expression and morphology of DRG neurons in culture**. (A&B) Small diameter DRG neurons in culture express the TrkA receptor and TRPM8 channels. Immunofluoresence images of 12 hr DRG cultures stained with (A) an anti-TrkA (green), (B) an anti-TRPM8 (green) antibodies, and DAPI (blue). Analysis of somatic diameters displaying immunoreactivity (closed circles) for (C) TrkA and (D) TRPM8 show a significant bias of TrkA and TRPM8 immunoreactivity (closed circles) in smaller neurons (<15 μm) compared with TrkA and TRPM8 negative neurons (open circles). (n = 326 and 223 cells counted respectively, scale bar is 20 μm). (E) TRPM8 immunoreactivity in culture. DRG neurons displaying effusive growth cone morphologies (suitable of calcium imaging experiments) were TRPM8 positive (scale bar is 50 μm).

These results raised the possibility that a TTR-induced influx of cations through TRPM8 channels may lead to a membrane depolarization that is sufficient to activate tetrodotoxin-resistant (TTX_R_) sodium channels, with the subsequent opening of VGCCs. To test this idea, we examined the effect of the TRPM8 agonist icilin (100 μM) on calcium influx. Icilin treatment caused a similar increase in [Ca^2+^]_i _to that of L55P (Figure 4B), suggesting that the majority of cultured neurons were TRPM8 positive. In contrast, the vanilloid receptor (TRPV) agonist capsaicin (1 μM) resulted in a significantly decreased response in growth cone [Ca^2+^]_i_. When DRG neuron cultures were pretreated with ambroxol (5 μM) for 10 min, the icilin-induced increase in [Ca^2+^]_i _was also significantly attenuated (Figure 4B). The presence of TRPM8 channels was confirmed by immunocytochemical staining (Figure 5B and 5D), which demonstrated that the majority (56%) of small-diameter neurons (i.e. neurons with a nuclear diameter <15 μm) in the culture were TRPM8 positive, and a similar percentage (48%) were TrkA positive (Figure 5A and 5C). Since previous studies have shown that TRPM8-positive neurons *in vivo *almost exclusively express TrkA [31], and our DRG culture medium contained NGF, which enhances cell survival and stimulates neurite outgrowth via a TrkA-dependent mechanism [36], it was not surprising that most of the surviving small diameter neurons with growth cones were TRPM8 positive (Figure 5E). Taken together, these results demonstrate that although TRPM8 channels are found only on a relatively small (13-20%) population of DRG neurons *in vivo *[31], they are a major TRP channel on DRG neurons cultured in the presence of NGF. Significantly, the possibility that TRPM8 conductance was sufficient to activate Na_V_1.8 channels was confirmed by the inhibition of the icilin-induced calcium entry in small diameter DRG neuron growth cones by a Na_V_1.8 antagonist, ambroxol.

### siRNA silencing of TRPM8

**Figure 6:**
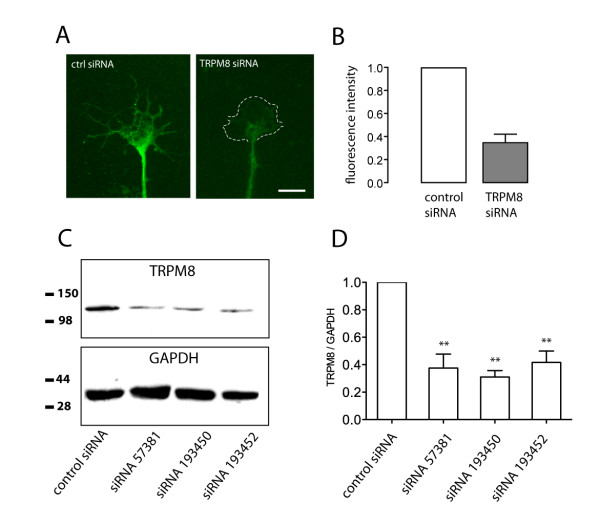
**siRNA silencing of TRPM8 expression**. Figure shows the level of TRPM8 immunoreactivity as determined by immunocytochemistry and by western blotting after treatment with siRNA oligonucleotides. (A) TRPM8 staining of a DRG growth cone treated with a non-specific control siRNA or with a specific TRPM8 siRNA oligonucleotide (#57381). The dotted line describes outline of the growth cone in the TRPM8 siRNA culture. (B) Quantitative analysis of TRPM8 immunocytochemistry shows that there was a significant decrease in TRPM8 expression in the presence of the TRPM8 siRNA (#57381, n = 73 growth cones) compared with the non-specific control siRNA (n = 56 growth cones). (C,D) Western blot and quantitation of TRPM8 immunoreactivity after treatment with 3 different TRPM8 siRNA oligonucleotides and a non-specific control siRNA. (C) Extracts from siRNA-treated DRG cultures were applied to SDS gels and probed with an anti-TRPM8 antibody. (D) Quantitative analysis of blots revealed significant knockdown of TRPM8 protein expression compared to cultures treated with a non-specific control siRNA. TRPM8 siRNA levels were normalised to GAPDH expression. Figure shows 60-70% knockdown achieved with 3 separate TRPM8 oligonucleotides. Values are means of 9 replicate immunoblot lanes over 3 separate experiments. Significant differences from control are depicted as: ** p < 0.005 as determined by a Mann-Whitney U-test. Error bars show means ± SEM

To confirm the involvement of TRPM8 channels in L55P-induced calcium entry, we used a siRNA knockdown strategy in combination with calcium imaging of DRG growth cones. DRG neurons were loaded with fluorescently (Cy3) tagged siRNA to identify those cells that had taken up the oligonucleotide. Immunostaining of control siRNA loaded DRG growth cones with a polyclonal anti-TRPM8 antibody revealed extensive, but non-homogeneous punctate staining throughout DRG growth cones, extending from the central area to distal filopodial tips (Figure 6A). In neurons loaded with a specific TRPM8 siRNA (#57381), there was significant knockdown of TRPM8 expression, especially in filipodial tips, lamellipodia and transitional zone of growth cones (Figure 6A,B). The specificity of knockdown was confirmed by western immunoblot analysis of DRG primary cultures loaded with control and specific TRPM8 siRNA oligonucleotides (Figure 6C,D). Proteins in DRG cultures were separated by SDS-PAGE, transferred to a PVDF membrane and probed with a polyclonal anti-TRPM8 antibody. A band of immunoreactive protein migrated at a molecular mass of 135 kDa, consistent with the predicted size of TRPM8 (Figure 6C). The staining intensity of the TRPM8 band was significantly reduced in cells loaded with TRPM8 siRNA oligonucleotides, when compared with cells treated with a control oligonucleotide (Figure 6D).

**Figure 7:**
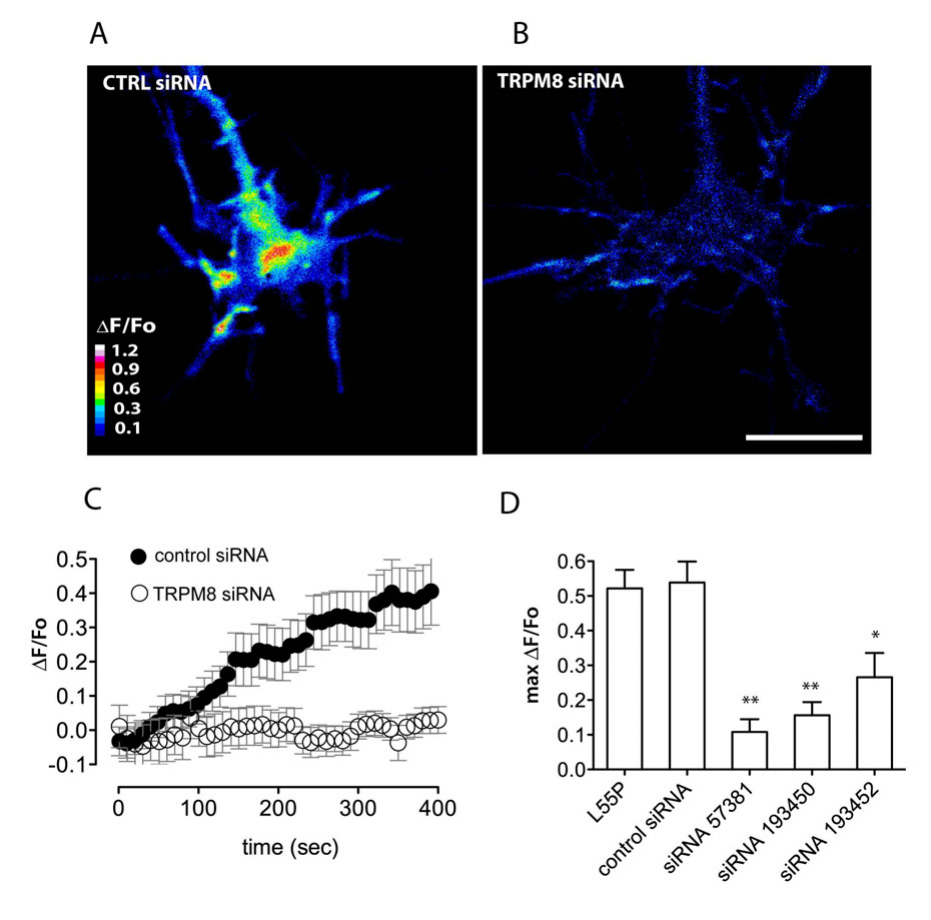
**Effect of TRPM8 knockdown on TTR-induced calcium influx**. TRPM8 channels are necessary for L55P-induced calcium influx. Representative images of DRG neurons loaded with (A) non-specific control siRNA elicited a greater L55P-induced calcium influx (ΔF/F_0_) than growth cones loaded with (B) specific TRPM8 siRNA (#57381). (C) Pooled results show calcium influx (ΔF/F_0_) in response to L55P for control siRNA (closed circles, n = 12) and a specific TRPM8 siRNA (open circles, n = 16) from 3 separate experiments. (D) Using 3 different TRPM8 siRNA oligonucleotides results in a similar reduction in L55P-mediated calcium entry. Significant differences from control are depicted as: *p < 0.05; **p < 0.005; Mann-Whitney U-test. Error bars indicate mean ± SEM. Scale bar is 5 μm.

To determine the effect of TRPM8 knockdown on L55P-induced calcium influx, DRG neurons were treated with each of three separate TRPM8 siRNA oligonucleotides and then examined for L55P-induced calcium entry using calcium imaging (Figure 7A,B). Significantly, there was almost complete abolition of the calcium influx effect in the TRPM8 siRNA treated neurons, whereas neurons treated with a non-specific control siRNA responded to the TTR treatment with an increase in [Ca^2+^]_i _(Figure 7C,D). The data from the pharmacological studies and from the siRNA studies, when taken together, clearly demonstrated that TRPM8 channels are required for L55P-induced calcium entry in DRG growth cones.

## Discussion

In this study we report that the mutant TTR variant L55P, induces calcium influx in growth cones of DRG sensory neurons. The extracellular calcium influx seen in DRG growth cones occurred within minutes of exposure to L55P and was sustained for at least 500 sec. The observation that L55P-mediated increases in growth cone calcium originated exclusively from extracellular sources confirms previous work in SH-SY5Y cells that showed TTR-induced calcium influx is mediated primarily by ion channels at the plasma membrane [12]. In FAP, deposition of aggregated TTR protein is a pathological hallmark of a polyneuropathy and subsequent peripheral neurodegeneration. These results strongly suggest that in FAP, distal sensory neurons are exposed to prolonged calcium dysregulation, leading to progressively aberrant signalling in small-diameter sensory neurons.

Our data show that a significant portion of the calcium influx was due to the opening of VGCCs. For example, approximately 50% of the calcium influx was blocked by the L-type channel blocker nifedipine. We considered whether voltage-gated sodium channel activity was responsible for the opening of VGCCs. To answer this question, we examined the effect of voltage-gated sodium channel blockers on TTR-induced calcium influx. Nociceptive sensory neurons express a subset of Na_v _channels that are resistant to TTX blockade [30,37-40]. Na_v_1.8 channels in DRGs are activated by protein kinase A (PKA) and protein kinase C (PKC) dependent mechanisms and they are involved in diabetes-induced hyperalgesia and allodynia where they exhibit significantly increased slow and fast ramp activation profiles and left-shifted voltage-dependent activation [41,42]. Consistent with the possibility that voltage-gated sodium channels mediate the TTR-induced opening of VGCC, we found that while TTX had no significant effect on calcium influx, ambroxol and carbamazepine, blockers of TTX_R _Na_v_1.8 channels [43,44], inhibited up to 80% of the calcium influx. As a small percentage of the calcium influx was not inhibited by ambroxol or carbamazepine, this suggested that some influx may have occurred via a voltage-insensitive mechanism. This latter possibility was supported by the finding that SKF96365, a relatively non-specific inhibitor of many types of TRP channels (including TRPM8 channels) [33,34], and BCTC, an inhibitor of TRPM8 and TRPV1 channels [35] significantly inhibited calcium influx. The involvement of TRPM8 channels in TTR-induced calcium influx was confirmed by experiments which showed that the TRPM8 agonist icilin induced similar calcium influx in DRG growth cones and that the effect of TTR was strongly inhibited by three different TRPM8 siRNA oligonucleotides. As TRPM8 channels are permeable to both sodium and calcium [45,46], it seems likely that they are both directly responsible for some of the calcium influx, but they may also contribute to the changes in membrane potential which trigger opening of voltage-gated sodium and calcium channels.

It was surprising to observe that TRPM8 channels were responsible for calcium influx in most growth cones. Previous studies have shown that TRPM8-positive cells account for only a small fraction of the total population of neurons in DRG tissue [45,46]. However, in our DRG cultures, TRPM8-positive cells accounted for >50% of all neurons. The greater percentage of TRPM8-positive cells in the cultures compared with the number *in vivo *is likely to be due to the positive selection of TrkA-positive small diameter neurons since the cells were cultured in the presence of NGF.

TRPM8 channels have been described as the prototypic thermosensitive ion channel [45,46]. They are activated by the compounds menthol, icilin and mild cold stimuli and are a prominent receptor subtype on small diameter (C and Aδ fibre) DRG neurons [47]. While FAP exhibits a variety of clinical manifestations, one of the most common symptoms is a progressive parasthesia involving the extremities, especially the lower limbs and affecting thermosensation and nociception [6,7,48-50]. While the current study strongly suggests that TRPM8 and Na_v_1.8 channel activation occurs in response to L55P attack on DRG neurons *in vitro*, the involvement of other ion channels in TTR toxicity cannot be excluded. However, it is likely the parasthesia seen in FAP is a clinical correlate of a small diameter sensory neuron phenotype undergoing chronic excitotoxic insult.

The precise TTR species that mediate the effect on calcium influx are yet to be elucidated. Preparations of L55P contained significant amounts of oligomeric aggregates (~1800 kDa) that were not seen in preparations of wild-type TTR. Urea-denaturation studies suggest that unlike WT TTR, L55P aggregates through a complicated pathway involving the formation of dimers and trimers. However the precise mechanism responsible for the formation of soluble oligomers at physiological pH has yet to be determined [14]. Soluble oligomeric TTR aggregates are more toxic to cells than fibrillar or monomeric species [12,16]. We have previously shown that the aggregation state of TTR variants is correlated with the magnitude of calcium entry and cytotoxicity in SH-SY5Y cells [12]. In the present study, freshly prepared L55P elicited a greater calcium influx in DRG growth cones than either V30M or WT proteins. Reixach *et al*. [51] have reported that while monomers and small oligomeric (<100 kDa) forms of the V30M variant induced cell death in a human neuroblastoma cell line, the formation of these smaller species proceeded at a relatively slow rate under physiological conditions.

It remains unclear how aggregated TTR can activate TRPM8 channels. One possibility is that TTR binds directly to the channels and causes a conformational change which leads to channel opening. However, there is no direct evidence for this. Another possibility is that TTR binds to a membrane component which triggers the opening of TRPM8 channels. This membrane component could be a lipid. TTR binds preferentially to phospholipids in cholesterol-rich regions of membranes and alters the membrane fluidity [13]. There is some evidence of preferential localisation of TRPM8 activity at detergent-insoluble areas of the plasma membrane or lipid rafts [52,53]. As TRPM8 channels are mechanosensitive [54-58], it is possible that disruption of the lipid membrane by TTR could contribute to channel opening. Also, like other TRPM family members, TRPM8 is activated by a major phospholipid component of lipid rafts, phosphatidylinositol 4,5-bisphosphate (PIP_2_) [59-61]. It is likely, therefore, that L55P-mediated calcium influx at the cell membrane is the culmination of multiple interactions and further work will be required to fully explore this possibility.

The present study identifies TRPM8 channels as a possible molecular target for pharmacological intervention in FAP. Currently, liver transplantation is an effective treatment for FAP, and several pharmacological interventions such as clonazepam, doxepine and significantly, carbamazepine, have been shown to be effective in the symptomatic treatment of the polyneuropathy and parasthesia associated with FAP [48]. Recently, small molecule stabilisers which inhibit the aggregation of TTR have been undergoing promising clinical trials [20],.

As amyloidoses may share common mechanisms of neurotoxicity, this study has implications for understanding the cause of other neurodegenerative diseases. Neuronal calcium dysregulation has also been implicated in the pathogenesis of a variety of amyloidoses, including AD [62,63]. Accumulation of aggregated Aβ in the AD brain has been reported to disrupt calcium metabolism with concomitant downstream cytotoxic effects such as mitochondrial dysfunction, activation of caspases [64,65] microtubule destabilisation and tau phosphorylation [66]. While there is some evidence implicating TRP channels in the pathogenesis of AD [67], it is unclear whether aggregated Aβ interacts directly with TRP channels in the AD brain and therefore future experiments in this area would add considerably to our understanding of the early neurotoxic events in AD.

## Conclusions

In this study, we examined the mechanism of TTR-induced calcium influx in sensory neuron growth cones. We show that an amyloidogenic form of TTR, L55P, elicited an extracellular calcium influx in a process mediated by TRPM8 and Na_v_1.8 channels. Our findings suggest a molecular correlate of FAP pathogenesis involving calcium dysregulation at the cell membrane caused by aggregated or misfolded TTR proteins.

## Methods

### Materials

Wild-type (WT), V30M and L55P transthyretin were expressed in BL21(DE3)- RIG CodonPlus E.coli and purified as described previously [13]. Proteins were incubated for2-4 hr at 22-24°C prior to their use in calcium imaging experiments. Pharmacological compounds used in experiments were obtained from the following sources: nifedipine, ω-agatoxin IVA, ω-conotoxin GIVA (Alomone Labs, Israel), SKF-96365 (Tocris, Bristol, UK), [N-(4-tert-butylphenyl)-4-(3-chloropyridin-2-yl)piperazine-1-carboxamide] (BCTC), (ENZO, NY, USA), ambroxol and carbamazepine (Sigma-Aldrich, MO, USA).

### Cell culture

All animal experimentation was performed in accordance with the guidelines and protocols of the Animal Ethics Committee, University of Tasmania. Dissected thoracic dorsal root ganglia from embryonic day 16-18 (E16-18) hooded Wistar rats were mechanically dissociated into sensory neuron medium (SNM) composed of Dulbecco's modified Eagle's medium/Ham's F-12 medium 1:1, (Sigma-Aldrich, MO, USA) containing foetal bovine serum (5% v/v), penicillin G (100 U/ml), streptomycin (100 μg/ml), nerve growth factor (NGF, 50 ng/ml, Sigma-Aldrich, MO, USA) and N2 neural medium supplement (Invitrogen, CA, USA). Cells were plated onto poly-ornithine (100 μg/ml, Sigma, MO, USA) and laminin (50 μg/ml, Invitrogen, CA, USA) coated glass coverslips.

### Calcium imaging

Between 12 and 24 hr after plating, sensory neurons, grown on coverslips, were loaded with Fluo-4 AM calcium indicator (1 μM, Invitrogen, CA, USA) in Hanks balanced salt solution (HBSS) without phenol red (Sigma-Aldrich, MO, USA) for 7 min, rinsed with fresh HBSS and incubated for a further 15 min at 37°C to allow complete de-esterification. All imaging experiments were carried out at 24-26°C. Calcium-free experiments were performed in calcium-free HBSS (Sigma-Aldrich, MO, USA) supplemented with 300 μM EGTA (glycol-bis(2-aminoethylether)-N,N,N',N'-tetra-acetic acid). Growth cones from small neurons (<15 μm in diameter) were selected for analysis. Freshly prepared TTR proteins were added to culture dishes to a final concentration of 0.5 mg/ml for all experiments and remained in contact with growth cones for the duration of the imaging period. Calcium experiments on aged (36 hr) protein were only performed using the V30M variant. Fluorescence images were acquired at 1-5 Hz using a confocal microscope (Zeiss LSM 510, Jena, Germany) using a 20× (1.0 NA) water immersion objective. Fluorescence images and pixel intensities were analysed using ImageJ software [68] and custom software (Matlab, MathWorks, MA, USA). ΔF for each image frame was calculated by subtracting integrated pixel intensity of a region of interest encompassing the entire growth cone from the average integrated pixel intensity or F_0_, of the first 20-30 frames of the imaging session for each cell. Maximal ΔF/F_0 _was calculated for each growth cone over the entire 7 min imaging period. Prism 4 (GraphPad Software, CA, USA) and Adobe Illustrator CS3 (Adobe Systems, CA, USA) were used for graphical and statistical analysis of data.

### Atomic force microscopy (AFM)

AFM was performed as previously described [12]. Briefly, TTR solutions (5-50 μg/mL), were deposited on a surface of freshly cleaved, highly oriented pyrolytic graphite and incubated at 37°C for 30 min. AFM analysis was performed on a Digital Instruments Nano-Scope IV, multimode scanning probe microscope equipped with a 15 μm E scanner (Veeco Instruments, NY, USA). Images were obtained in tapping mode at oscillation frequencies of 200-300 kHz and analyzed using WSxM 4.0 software (Nanotec Electronica S.L., Madrid, Spain).

### Analysis of TTR aggregation by dynamic light scattering (DLS)

Aggregation of wild-type (WT) and L55P TTR (1 mg/mL in 20 mM phosphate buffer (pH 7.4) containing 150 mM NaCl) was measured at 37°C for 6 hr using DLS. Measurements at 30 min intervals were acquired on a Zetasizer Nano S (Malvern Instruments, UK), and analyzed using Zetasizer Nano software 5.0.2. The hydrodynamic diameter and predicted molecular masses of protein species were calculated using Zetasizer Nano software 5.0.2, utilising the Stokes-Einstein equation and the Perrin factor to derive an estimate of molecular mass for a globular protein [69].

### siRNA knockdown of TRPM8 expression

Fluorescently (Cy-3) labelled TRPM8 siRNA (GGCCAUGGAGAGCAUAUGC), (CGAGAAUGCGUCUUCUUUA), (GCACAAAAAUGUAUGGAAA) and negative control siRNA oligonucleotides were obtained from (Applied Biosystems, CA, USA). Oligonucleotides were loaded into sensory neurons using previously established methods [70-72]. Briefly, oligonucleotides (5 nM) were added to dissected DRGs in SNM. DRGs were then gently triturated and maintained in SNM (with 5 nM oligonucleotide) throughout the culture period (24 hr). Incorporation of fluorescently labelled oligonucleotides was confirmed by confocal microscopy (data not shown).

### Immunocytochemistry

DRG cultures were fixed in 4% (w/v) paraformaldehyde and blocked with 10% (v/v) goat serum for 4 hr at 4°C. The primary antibodies, a polyclonal rabbit anti-TRPM8 (extracellular) (1:1000, Alomone Labs, Israel) or polyclonal rabbit anti-TrkA (1:1000, Abcam, Cambridge, UK) were added to coverslips and incubated for 4 hr at 22°C. Detection of primary antibodies was performed using fluorescently labelled goat anti-rabbit antibodies (Invitrogen, CA, USA). Nuclear staining was performed with 4',6-diamidino-2-phenylindole (DAPI) (Invitrogen, CA, USA). Images were acquired on a Zeiss LSM 510 confocal microscope and processed using ImageJ, Adobe Photoshop CS3 and Adobe Illustrator CS3 (Adobe Systems, CA, USA).

### Immunoblotting

Primary DRG cultures treated with either control or specific TRPM8 siRNA oligo (5 nM) were established at high density in 6-well polystyrene culture plates (BD Falcon, NSW, Australia). Following incubation for 24 hr, cells were rinsed 3 times in ice-cold PBS. Ice-cold cell lysis buffer (50 mM Tris-HCl pH 7.4, 150 mM NaCl, 1 mM EDTA, 1%, v/v, Triton X-100, 1%, w/v, Na deoxycholate, 0.1%, w/v, SDS) supplemented with Complete^® ^Mini protease inhibitor cocktail (Roche Diagnostics, NSW, Australia) was added to dishes and slowly agitated for 15 min at 4°C. Cell lysates (10 μg total protein) were separated on 8% SDS-PAGE, transferred onto PVDF membranes and then blocked overnight in blocking solution [5% (w/v), non-fat skim milk in 20 mM Tris-HCl and 150 mM NaCl (TBS)]. Primary antibodies, a rabbit anti-TRPM8 (1:200 in blocking solution, Alomone Labs, Israel) and mouse anti-GAPDH (1:2000 in blocking solution, Sigma-Aldrich, MO, USA) were added to the membrane, incubated overnight at 4°C then rinsed thoroughly in TBS. Membranes were subsequently probed with goat anti-mouse-HRP or goat anti-rabbit-HRP secondary antibodies for 1 hr at room temperature. Conjugates were detected using Immobilon chemiluminescent substrate reagent (Millipore, MA, USA) and a Chemi-Smart 5000 CCD image acquisition system (Vilber Lourmat, Marne-la-Vallee, France). CCD image analysis was performed with ImageJ software and statistical analysis was performed with Prism 4 (GraphPad Software, CA, USA).

## Competing interests

The authors declare that they have no competing interests.

## Authors' contributions

RG carried out the imaging, DLS and knockdown experiments, analysed and interpreted the data, participated in overall direction of the study and preparation of the manuscript. XH carried out the AFM experiments. DK participated in the analysis and interpretation of the DLS experiments. HC, AV, LF and HP were involved in the analysis and interpretation of data and critical revision of the manuscript. DS conceived and participated in overall direction of the study and preparation of the manuscript. All authors have read and approved the final manuscript.

## Supplementary Material

Additional file 1 **Comparison of aged and fresh V30M protein on DRG calcium influx**. V30M protein was aged for 36 hr at 24°C then added to DRG cultures (0.5 mg/ml) in the calcium imaging assay as described (n = 4). Aged V30M elicited a smaller calcium influx than freshly prepared L55P protein, but a greater calcium influx than freshly prepared V30M protein. Click here for file
