# Supplementary figures and images for: TRPM8 and Nav1.8 sodium channels are required for transthyretin-induced calcium influx in growth cones of small-diameter TrkA-positive sensory neurons
Source: Mol Neurodegener. 2011 Mar 4;6:19. doi: 10.1186/1750-1326-6-19 (PMC3058062; doi:10.1186/1750-1326-6-19)

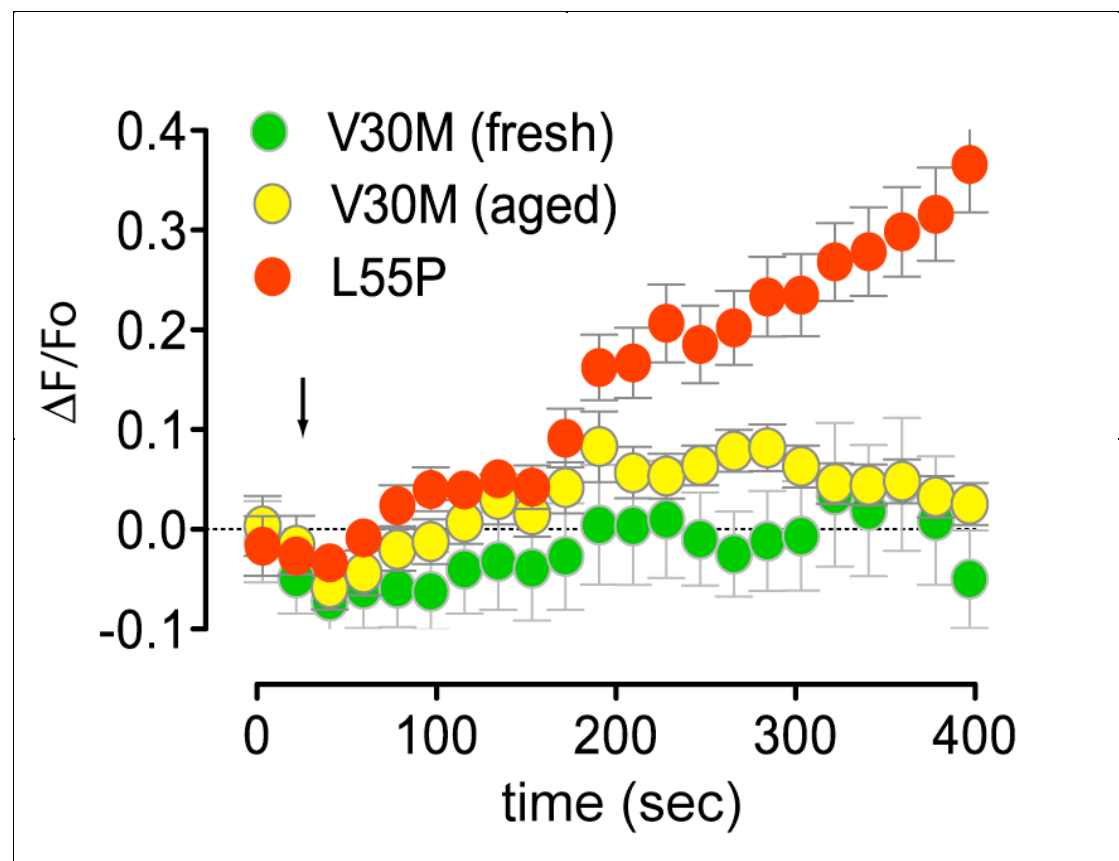

Supplement: Additional file 1 — Comparison of aged and fresh V30M protein on DRG calcium influx. V30M protein was aged for 36 hr at 24°C then added to DRG cultures (0.5 mg/ml) in the calcium imaging assay as described (n = 4). Aged V30M elicited a smaller calcium influx than freshly prepared L55P protein, but a greater calcium influx than freshly prepared V30M protein. [file 1750-1326-6-19-S1.PDF]
